# Translation, Adaptation, and Validation of the Malay Version of the Barriers to Access to Care Questionnaire for Assessing the Barriers to Seeking Mental Health Care Among the Health Workforce in the East Coast Region of Peninsular Malaysia

**DOI:** 10.7759/cureus.41405

**Published:** 2023-07-05

**Authors:** Muhammad S Kunyahamu, Aziah Daud, Tengku A Tengku Ismail, Mohd F Md Tahir

**Affiliations:** 1 Department of Community Medicine, Universiti Sains Malaysia School of Medical Sciences, Kota Bharu, MYS; 2 Department of Psychiatry and Mental Health, International Islamic University Malaysia, Kuantan, MYS

**Keywords:** bace3, malay, questionnaire validation, health workforce, mental health, questionnaire translation and adaptation, help seeking barriers

## Abstract

Background

Mental health problems among the health workforce are a significant concern worldwide, including in Malaysia. Unfortunately, some health workforce may perceive various barriers or challenges that prevent them from seeking help. Identifying and addressing these barriers is crucial for enhancing mental health services and support. The Barriers to Access to Care Evaluation (BACE-3) questionnaire is a valuable tool that can be used for assessing these barriers among health workers. However, a validated Malay version is needed. Therefore, this study aims to translate, adapt, and validate the original version of Barriers to Access to Care Evaluation (BACE-3) into the Malay version (MBACE).

Methods

A rigorous process of translation and adaptation was followed to develop the Malay version of the BACE-3 questionnaire (MBACE). A cross-sectional study was conducted to assess the psychometric properties of the questionnaire, with purposive sampling employed to recruit 188 participants from various job categories, including doctors, nurses, pharmacists, and non-clinical staff, such as health assistants and clerks. The analysis was conducted using the R software version 4.2.2 (R Foundation, Vienna, Austria). Construct validity was determined using confirmatory factor analysis (CFA). To assess the convergent validity, internal consistency, and reliability of the instrument, measures such as the average variance extracted (AVE), composite reliability (CR), and Cronbach's alpha values were calculated.

Results

During the CFA process, two items with a factor loading less than 0.5 (items 15 and 16) were removed to improve the convergent validity and model fit. The CFA results revealed that the 2-factor model MBACE had good construct validity (root mean square error of approximation (RMSEA) = 0.053; comparative fit index (CFI) = 0.939; Tucker-Lewis fit index (TLI) = 0.934). The internal consistency was supported by Cronbach's alpha values ranging from 0.92 to 0.94 for the stigma factor and non-stigma factor. The average variance extracted (AVE) and composite reliability (CR) values further supported the questionnaire's reliability and convergent validity.

Conclusion

The translated and adapted 28-item MBACE questionnaire is a valid and reliable tool for assessing the barrier to seeking professional mental health care among the Malaysian health workforce. This instrument has the potential to aid in the development of targeted interventions to promote mental health help-seeking behavior and enhance the well-being of the Malaysian health workforce.

## Introduction

Mental health problems are conditions that affect a person's mood, thinking, and behavior, leading to distress and reduced ability to function. They can range from mild stress to more severe and disabling conditions, such as schizophrenia and bipolar disorder [1]. A person's quality of life can be affected by mental health problems, which can lead to disability, social exclusion, or even suicide if not properly addressed. Mental health problems are a significant global public health concern, affecting many people, including those who work in the health sector.

Many studies have indicated that the health workforce may experience higher rates of certain mental health problems compared to the general population, potentially influenced by a variety of factors, including the nature of their work. In recent years, the impact of mental health problems in the health workforce has been recognized as a significant matter of concern [2, 3]. In response to this situation, the United Nations has highlighted the mental health needs of the health workforce as a global priority [4]. In relation to the Malaysian health workforce, a press statement by the Ministry of Health in September 2021 indicated that about 14.2% of the health workforce suffered from severe mental health problems [5]. This suggests that issues with mental health are a matter of concern among the Malaysian health workforce.

Taking the initiative to seek help from a mental health professional, such as a counselor, psychologist, doctor, or psychiatrist, can be a crucial first move toward recovery. However, despite the benefits of obtaining professional help for mental health problems, several barriers impede individuals from seeking such help, and this also occurs among people who work in the health sector, even though they work in the sector that provides mental health support services. Some of these barriers include stigma and fear of being judged or discriminated against by others, concerns about career progression, concerns about confidentiality and privacy, lack of time, and poor attitudes, such as the thought of being capable of managing their own mental health [6-9].

It is important to address these barriers and work towards improving access to mental health services for those who need them. To tackle these issues among the health workforce, a valid and reliable assessment tool is needed to identify the relevant factors that can act as barriers. Currently, the Barriers to Access to Care Evaluation (BACE-3) questionnaire is among the available and validated tools that have been used to assess barriers to seeking help for mental health problems. Based on current evidence, BACE-3 stands out as an instrument that is reliable, simple to use, user-friendly, and possesses good psychometric properties [10].

Since BACE-3 originates from Western countries, it must be translated and adapted to Malaysian culture, which differs from Western culture. In Malaysia, Malay is the official language in use. This makes the original version of the questionnaire not widely applicable for use in Malaysia. Additionally, cultural and linguistic differences can affect the validity and reliability of the questionnaire, making it necessary to adapt and validate them in different cultural and linguistic contexts suitable to the targeted audience [11].

As far as the authors are aware, there have been no previous attempts to translate the BACE-3 into Malay, and no reliable and valid tool has been specifically developed to assess the barriers faced by Malaysian health workforces when seeking professional mental health services. Given the importance of having a local language version of this questionnaire, therefore, this study aims to translate, adapt, and validate the Malay version of BACE-3 (MBACE) so that the barriers to seeking professional help for mental health can be accurately assessed among the Malaysian health workforce.

## Materials and methods

Ethical clearance

This study received approval from the Malaysia Research Ethics Committee (MREC) of the Ministry of Health Malaysia (NMRR ID-22-01529-H11) and the Human Research Ethics Committee, USM, Malaysia (USM/JEPeM/22070479) and was conducted in accordance with the Declaration of Helsinki of 1975, as revised in 2013. Informed consent was obtained from every respondent. The respondents were provided with a comprehensive understanding of the research process and had the option to withdraw at any point. Participants' information was kept strictly confidential, with only the researchers having complete access to the data.

Barriers to Access to Care Evaluation (BACE-3)

In 2012, BACE-3 was designed in the Health Services and Population Research Department of the Institute of Psychiatry at King's College in London, England by Clement et al. [10], in light of the fact that there should be a more comprehensive measure of the barriers that people could face when trying to get the professional help they need for their mental health. It was specifically designed to assess the extent to which the items listed can act as obstacles to seeking help from mental health professionals. It consists of 30 questions scored on a three-point Likert scale. A score of zero indicates "not at all", while a score of three indicates "a lot". Thus, a higher score represents a greater barrier.

The BACE-3 was developed through a thorough and rigorous process that involved an extensive review of the existing literature. Potential barrier items were then thoroughly evaluated against specific criteria and systematically reduced from 172 items to a final set of 30. Basically, BACE-3 can be divided into two main domains (stigma barrier and non-stigma barrier). The tool was tested by Clement et al. [10], who found it to display good internal consistency, test-retest reliability, and both content and construct validity. The tool was also found easy to understand by children aged between 11 and 12 years old [10]. This meant that it was easier for respondents to complete the questionnaire, which is an important key consideration for any survey that hopes to yield a good response rate [12]. The tool has been widely used in research to assess actual and perceived barriers to professional mental health care among individuals with or without mental illness [8-10, 13, 14]. This tool has since been translated into several languages, including Japanese [15], Portuguese [16], Spanish [17], and Telegu [18]. It is important to note that specific versions of the Portuguese and Spanish questionnaires have been adapted to the unique cultural contexts of Brazilian [19] and Colombian [20], respectively.

Translation and adaptation

All 30 items were translated into Malay according to international guidelines as specified by Wild et al. [21] after obtaining permission from the original authors of the questionnaire. This process began with the forward translation of the questionnaire from English to Malay by two translators who were fluent in both Malay and English. They were blinded to the original questionnaire and the objectives of the study. Each translator produced a Malay version of the questionnaire without mutual consultation. They were required to translate with terminology or language that was easily understood by the public. 

The next step was the reconciliation of the translation. The objective of the reconciliation was to compare and combine both forward translations into a single forward translation. Two versions of the translations thus allowed the researchers to compare any variations or divergences in the translation, hence reducing the likelihood of bias. Any discrepancy between the translated versions was discussed among the research team and a reconciled Malay version that most closely matched the original was sought.

The reconciled Malay version was then back-translated into English by two translators who were fluent in English and Malay. They were also blinded to the original questionnaire and the objectives of the study. The back-translated version was subsequently compared to the original version. This was done to ensure conceptual parity with the original document. Word selections that differed between the original and the backward translations were reviewed until the preliminary Malay version was finalized to ensure that the questionnaire was understandable and relevant to the audience for whom it was intended.

The preliminary Malay version of the questionnaire's content validity was then assessed by a panel of experts to quantify its content validity. Each expert was required to determine the relevancy of each item in assessing barriers to seeking help for mental health using a four-point ordinal rating scale, where one indicated that the item was not relevant or appropriate and four indicated that the item was highly relevant and appropriate. The content validity index (CVI) was assessed by calculating both the item level CVI (I-CVI) and the scale level CVI (S-CVI) [22]. The I-CVI was computed for each item by dividing the number of experts who rated the item as three or four by the total number of experts who assessed the items. The S-CVI was computed by dividing the number of items that received a rating of three or four by all the experts by the total number of items. Only items with an I-CVI greater than 0.83 and an S-CVI greater than 0.9 were retained in the instrument [22].

**Figure 1:**
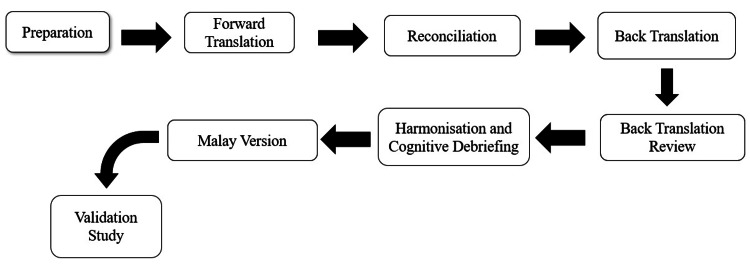
Flowchart of the study process, from initial translation to final validation of the questionnaire

Face validation (FV) testing was subsequently carried out with a sample of 10 health workers, who were carefully selected to represent the targeted population. They were recruited after taking into consideration a few factors, such as professional background, experience, and demographic characteristics relevant to the target population. The aim of this testing was to ascertain, from the perspective of the respondents, that the items and the instrument were valid. Any inappropriate items or other issues that may cause confusion were highlighted in this step. All respondents were asked to assess the comprehensibility and clarity of the translated items in the questionnaire using a four-point ordinal rating scale (1 = not clear and not understandable, 2 = somewhat clear and understandable, 3 = quite clear and understandable, and 4 = very clear and understandable). The face validity index (FVI) was used to quantify the FV. The FVI was calculated for each item by dividing the number of respondents who rated the item as three or four by the total number of respondents assessing the items. An FVI cut-off value of 0.8 was adopted, in line with recommendations in the literature [23]. The researchers then analyzed the results of content validation and face validation and constructed the final Malay version of the BACE-3 questionnaire, which was then assessed for reliability and validity among the targeted group population. Figure 1 provides a clear overview of the process, outlining the different phases of the study, from the first translation of the questionnaire to its final validation.

Psychometric testing of the Malay version of the questionnaire

A cross-sectional study was conducted from December 2022 to January 2023 to perform psychometric validation of the questionnaire using confirmatory factor analysis (CFA). Respondents were purposively recruited among the health workforce in one of the districts in the east coast region of Peninsular Malaysia. Purposive sampling was employed to select health workers from different job categories, such as doctors, nurses, pharmacists, and other health workers, including non-clinical workers, aiming to achieve a diverse sample by including individuals with various experiences and work settings within the healthcare industry. This sampling method facilitated the collection of information from a diverse group of health workers.

For this cross-sectional study, the minimum sample size was based on Costello and Osborne's [24] recommendations that for CFA, there should be at least five respondents for each item in the instrument being used; thus, 188 workers who fulfilled the study criteria were recruited. The inclusion criteria were workers aged >18 years with at least one year of working experience and able to read and understand the Malay language. Any workers with any conditions that prevented understanding or completion of the questionnaires were excluded from the study. Local health authorities were consulted to identify potential participants. Those who agreed to participate after being informed about the study's objectives and confidentiality measures were included in the sample. Participants were asked to personally fill out the questionnaire, which took between five to 10 minutes to complete. The questionnaires were distributed in paper format, and if respondents had any questions or concerns during the process, they were encouraged to seek clarification from the research team. Additionally, a brief demographic questionnaire was included.

Statistical analysis

R software version 4.2.2 (R Foundation, Vienna, Austria) was used in the analysis of the data. The construct validity of the tool was evaluated using CFA with the aid of "semTools" and "lavaan" packages available in the R software [25]. Given that the data were not distributed normally, robust maximum likelihood was preferred [26-28]. The goodness of fit of the model was assessed based on the following fit indices cut-off values: the root mean square error of approximation (RMSEA) <0.08, comparative fit index (CFI) ≥ 0.90, and Tucker-Lewis fit index (TLI) of >0.90 [27-29]. Based on the factor loadings, the model was revised. After measuring standardized factor loadings, all items that had factor loadings <0.5 were removed. The average variance extracted (AVE), composite reliability (CR), and Cronbach's alpha values were calculated to assess the convergent validity, internal consistency, and reliability of the questionnaire, respectively. As proposed by Bagozzi and Yi [30] and Hair et al. [27], AVE values above 0.5, CR values above 0.6, and Cronbach's alpha >0.7 indicate good reliability, internal consistency, and validity of the reflective construct. The model was then checked for multicollinearity. Multicollinearity exists if the inter-factor correlation ≥0.85 [28].

## Results

Content validation and face validation

All 30 items on the Malay version of the questionnaire achieved a rating of three or four by a panel of experts. The I-CVI values were calculated for each of the 30 items on the scale. The results showed that all items had an I-CVI of one. The S-CVI value was also calculated by averaging the I-CVI values across all items in the scale. The overall S-CVI was one. These findings indicate good content validity. For the face validity of the questionnaire, the FVI score was 1.00. Thus, none of the items were excluded from the final translated version of the questionnaire. On average, the time required to finish answering the Malay version of the questionnaire was roughly around seven minutes. The participants did not require any assistance in answering the questionnaire, and they verbally mentioned that they did not have any comments or suggestions to offer since they could understand the questions clearly.

General characteristics of the respondents

A total of 188 health workers agreed to participate in this study and returned a completed questionnaire. The mean age of the respondents was 37.64 years. Most of the respondents were female (73.9%) and Malay (95.7%). Table 1 shows the characteristics of the 188 respondents in this validation study who completed the questionnaire.

**Table 1:** Descriptive characteristics of the respondents (n=188) RM - Ringgit Malaysia; ^a^ - Single or Separated

Variables	Number	Percentage
Age, years (mean age ±SD: 37.64 ±7.59 years)		
Gender		
Male	49	26.1
Female	139	73.9
Ethnicity		
Malay	180	95.7
Non-Malay	8	4.3
Education level		
Secondary	37	19.7
Tertiary	151	80.3
Monthly income		
Less than RM 4850	165	87.8
RM 4850–10,959	22	11.7
>RM10,960	1	0.5
Marital status		
Married	169	89.9
Unmarried ^a^	19	10.1

Confirmatory factor analysis

There were no missing data. Based on the two-factor model (stigma factor and non-stigma factor), CFA was conducted to evaluate the construct validity of the model (model 1). Due to the multivariate non-normality of these data (kurtosis >5, p <0.05), the CFA was carried out using a robust maximum likelihood (MLR) estimate method. The following fit indices were found from the analysis of model 1: RMSEA = 0.051; CFI = 0.930; and TLI = 0.925. The standardized factor loading of each item ranged from 0.29 to 0.81. From these results, Model 1 generally had reasonable fit indices. However, the poor factor loading of items 15 and 16 with factor loadings of 0.29 and 0.34, which is less than the acceptable limit (FL³ 0.5) [27, 28] affected the convergent validity of model 1. Thus, a new model (model 2) was created by removing the items with a factor loading of <0.5 (Q15 and Q16). The fit indices of model 2 were recalculated, and the following was found from the analysis of model 2: RMSEA = 0.053; CFI = 0.939; TLI = 0.934 (Table 2). There was no evidence of multicollinearity between the items. For the assessment of the convergent validity, internal consistency, and reliability of model 2, AVE, CR, and Cronbach's alpha were calculated. As shown in Table 3, the AVE, CR, and Cronbach's alpha satisfied the criteria proposed by Bagozzi and Yi [30] and Hair et al. [27]. 

**Table 2:** Fit indices of the models CFI - comparative fit index; TLI - Tucker Lewis fit index; RMSEA - root mean square error of approximation

	RMSEA (90% CI)	CFI	TLI
Model 1	0.051 (0.042 - 0.060)	0.930	0.925
Model 2	0.053 (0.044 - 0.062)	0.939	0.934

**Table 3:** CFA results, factor loadings, composite reliability, average variance extracted, and Cronbach's alpha for model 2 (n=188)

Factor	Item	Standardized factor loading (FL)	Composite reliability (CR)	Average variance extracted (AVE)	Cronbach's alpha
Stigma	Q3	0.70	0.923	0.502	0.921
	Q5	0.81			
	Q8	0.63			
	Q9	0.79			
	Q12	0.73			
	Q14	0.64			
	Q17	0.65			
	Q19	0.74			
	Q21	0.73			
	Q24	0.61			
	Q26	0.77			
	Q28	0.67			
Nonstigma	Q1	0.77	0.943	0.509	0.940
	Q2	0.69			
	Q4	0.81			
	Q6	0.68			
	Q7	0.69			
	Q10	0.65			
	Q11	0.72			
	Q13	0.68			
	Q18	0.73			
	Q20	0.70			
	Q22	0.62			
	Q23	0.68			
	Q25	0.77			
	Q27	0.72			
	Q29	0.69			
	Q30	0.79			

## Discussion

This is the first study we are aware of that describes the process of adapting and validating a Malay version of the questionnaire focused on barriers to getting help from mental health professionals. This study's results provide a valuable resource for future research in Malaysia and other countries where the Malay language is spoken. The translation process was done carefully to ensure the translated version is suitable to the target population while striving to maintain its original content and meaning. The results from the content and face validity analyses suggested that all questions were clearly interpretable by the respondents and properly reflected the constructs of interest.

CFA results suggested a valid factor structure. The questionnaire's structural validity was checked using the RMSEA, TLI, and CFI. The initial model (model 1) demonstrated an acceptable model fit with an RMSEA value of 0.051, which is below the recommended acceptable value of 0.08 [27-29]. The CFI and TLI values were 0.930 and 0.925, respectively, which are above the recommended threshold of 0.90 [27-29], showing that model 1 fits the data well. Unfortunately, when calculating the AVE and CR to assess the convergent validity of the questionnaire, the AVE score for model 1 (non-stigma factor) was below the recommended threshold of 0.5, indicating that the convergent validity of model 1 is suboptimal. Upon closer inspection, it was discovered that this occurred due to items 15 and 16 having a poor factor loading (<0. 5), which indicates that the items were weakly related to the construct being measured compared to other items in the questionnaire [27, 30]. These items pertained to the availability of professionals from one's own ethnic or cultural group and the state of being too unwell to ask for help. Given that our sample was predominantly female and Malay, reflecting the demographic distribution of the health workforce in the district where the study was conducted, these items may have been less relevant or applicable, leading to their poor factor loadings.

Therefore, to address this issue, a new model (model 2) was developed and tested by removing items 15 and 16. Removing these items resulted in a significant increase in the AVE score, which is now above the recommended threshold of 0.5 [30], indicating that the revised model exhibited good convergent validity. The CR for each factor was also above the recommended threshold of 0.6, indicating good evidence of internal consistency reliability. The fit indices of model 2 were then recalculated and showed a slight improvement, with an RMSEA of 0.053, CFI of 0.939, and TLI of 0.934. Overall, these results suggest that model 2 is a better fit for the data than model 1. These findings also indicate that the stigma and non-stigma two-factor models effectively measure the intended construct and have a good underlying structure. Our finding also aligns with a previous study by Hongo et al. [16], who translated BACE-3 into Japanese and also found that the two-factor models were effective in measuring stigma and non-stigma constructs. This provides additional evidence for the model's validity and applicability across different contexts and among diverse populations. This is important for future research, as it demonstrates that the questionnaire can be used with confidence to assess the targeted constructs.

Further, the overall Cronbach's alpha value for the 28-item of MBACE was 0.957, with 0.921 for the stigma factor and 0.940 for the non-stigma factor, which exceeds the commonly recommended cut-off value of 0.70 [27]. The values were also not much different from the original English version [10]. This proves the reliability and consistency of all the items in measuring the construct of interest. Additionally, our result is also comparable with previous research that used this questionnaire and found similarly high levels of internal consistency [13, 16-18, 20]. Overall, these results demonstrate that the MBACE questionnaire is valid and supports its use for measuring the intended construct. These findings also proved that the MBACE maintains the original questionnaire's internal consistency and reliability. These results highlight the significance of determining the convergent validity of a measurement model to ensure the accuracy and reliability of the constructs being measured. By removing items with poor factor loadings, the revised model was able to more accurately and reliably measure the targeted constructs. These findings have implications for future research, as researchers should be aware of the potential impact of items with poor factor loadings on the validity and reliability of measurement models.

However, our findings should be interpreted in the context of our sample population, which was predominantly female and Malay. While this reflects the demographic distribution of the health workforce in the district where the study was done, it may not perfectly represent the overall demography of the health workforce in Malaysia. We acknowledge that the two items removed from the questionnaire, while enhancing its specificity to our sample population, might hold theoretical importance and could be relevant in a broader or different context. Therefore, we suggest that future research in this area could consider including these two items (Q15 and Q16). We further recommend that future studies should comprehensively assess a more diverse sample, including workers from different sectors, socio-economic backgrounds, and cultural groups that better represent the multiracial population of Malaysia. This will help to improve the generalizability and usefulness of the questionnaire in assessing barriers to mental health care among individuals from various ethnic and cultural backgrounds.

## Conclusions

In conclusion, this study provided initial psychometric evidence for the MBACE, demonstrating it as a reliable and valid tool for assessing the barriers to seeking professional mental health care among the health workforce in the Malay-speaking population. This tool, which we have successfully translated and adapted, is capable of enhancing mental health services among the Malaysian health workforce. The insights gained can then be used for the development of effective, targeted interventions to overcome these challenges and ensure that health workers get the mental health support they need.
